# Sophoridine induces apoptosis and S phase arrest via ROS-dependent JNK and ERK activation in human pancreatic cancer cells

**DOI:** 10.1186/s13046-017-0590-5

**Published:** 2017-09-11

**Authors:** Zihang Xu, Fei Zhang, Chao Bai, Chao Yao, Hairong Zhong, Chunpu Zou, Xiao Chen

**Affiliations:** 1 School of Basic Medical Science, Shanghai University of Traditional Chinese Medicine, Shanghai, 201203 China; 2 Department of General Surgery, Xinhua Hospital affiliated to Shanghai Jiao Tong University School of Medicine, Shanghai, 200092 China; 3 Department of general surgery, First Hospital of Shanxi Medical University, No. 85 South of Jiefang road, Taiyuan, 030001 China

**Keywords:** Pancreatic cancer, Sophoridine, Apoptosis, ROS

## Abstract

**Background:**

Pancreatic cancer is generally acknowledged as the most common primary malignant tumor, and it is known to be resistant to conventional chemotherapy. Novel, selective antitumor agents are pressingly needed.

**Methods:**

CCK-8 and colony formation assay were used to investigate the cell growth. Flow cytometry analysis was used to evaluate the cell cycle and cell apoptosis. The peroxide-sensitive fluorescent probe DCFH-DA was used to measure the intracellular ROS levels. Western blot assay was used to detect the levels of cell cycle and apoptosis related proteins. Xenografts in nude mice were used to evaluate the effect of Sophoridine on pancreatic cancer cell in vivo.

**Results:**

Sophoridine killed cancer cells but had low cytotoxicity to normal cells. Pancreatic cancer cells were particularly sensitive. Sophoridine inhibited the proliferation of pancreatic cancer cells and induced cell cycle arrest at S phase and mitochondrial-related apoptosis. Moreover, Sophoridine induced a sustained activation of the phosphorylation of ERK and JNK. In addition, Sophoridine provoked the generation of reactive oxygen species (ROS) in pancreatic cancer cells. Finally, in vivo, Sophoridine suppressed tumor growth in mouse xenograft models.

**Conclusion:**

These findings suggest Sophoridine is promising to be a novel, potent and selective antitumor drug candidate for pancreatic cancer.

**Electronic supplementary material:**

The online version of this article (10.1186/s13046-017-0590-5) contains supplementary material, which is available to authorized users.

## Background

Pancreatic cancer is still one of the deadliest solid malignancies across the world at present. Moreover, it has the poorest prognosis of any major tumor type, with a pretty low 5-year survival rate of approximately 5% for decades [1–3]. In addition, the lack of early clinical symptoms makes the early detection difficult. The severe drug resistance, including intrinsic and acquired, is thought to be responsible for the limited the therapeutic efficacy. Therefore, new effective treatments and drugs are urgently needed in order to improve the clinical outcome of pancreatic cancer patients.

Traditional herbal agents, containing various biologically active natural compounds, are claimed to have impressive therapeutic efficacy with minimal adverse effects, which provides sources and platforms for developing first-line drugs [4–6]. Sophoridine is a quinolizidine alkaloid isolated from traditional Chinese herbs, which exists in the stems and leafs of Leguminous plant Sophora alopecuroides L., Euchresta japonica Benth., and the roots of Sophora alopecuroides Ait. Accumulating evidence demonstrates remarkable pharmacological effects of Sophoridine, including anti-inflammatory [7, 8], anti-virus [9] and anti-cancer effects [10]. Recently, Sophoridine and its derivatives have drawn more and more attention owing to their various strong anti-tumor effects [11, 12]. The underlying mechanism of its anti-tumor effects is that Sophoridine can increase intracellular ROS levels and induce apoptosis [13, 14]. Additionally, other studies showed that Sophoridine can suppress tumors by inhibiting the activity of ubiquitin-proteasome pathway [15].

Here, we demonstrated that pancreatic cancer cells show extra vulnerability to Sophoridine compared with those of other cancer types. We further showed that Sophoridine leads to the S phage cell cycle arrest of pancreatic cancer cells, and induces apoptosis mainly via MAPK signaling pathway blocking. Therefore Sophoridine can inhibit the growth of pancreatic cancer cells in vitro and reduce the volumn of xenograft tumors in vivo*.* Our study demonstrated the promising preclinical anti-tumor activities of Sophoridine in pancreatic cancer.

## Methods

### Drugs and reagents

Sophoridine was kindly provided by National Institute for the Control of Pharmaceutical and Biological Products. Its purity was at least 95% as determined by HPLC analysis. Rhodamine 123, Hoechst 33,342 and Cycloheximide (CHX) were obtained from Sigma-Aldrich (MO, USA). Annexin V/PI apoptosis kit and Cell Counting Kit-8 (CCK-8) were relatively purchased from Invitrogen (CA, USA) or Dojindo Laboratories (Japan). DC protein assay kits were purchased from Bio-Rad, and the enhanced chemiluminescence plus system was purchased from Amersham Pharmacia Biotech. The antibodies against Bax, Bad, Bcl-XL, Bcl-2, cleaved-caspase 3 (Asp175), PARP cyt C and GAPDH were purchased from Cell Signaling Technology (MA, USA). ERK, JUNK and p-38 antibodies were purchased from Santa Cruz. Antibodies against Cyclin A, CDK2 and Cyclin D1 were purchased from Epitomics (CA, USA). PCNA antibody was obtained from Abcam (Cambridge, UK).

### Cell lines and cell cultures

The cell lines Miapaca-2, PANC-1 and HPDE were purchased from the Cell Bank of Type Culture Collection of the Chinese Academy of Sciences (Shanghai, China). Miapaca-2 cells were maintained in RPMI-1640 (Gibco, NY, USA) containing with 100 U/mL penicillin-streptomycin (Hyclone, UT, USA) and 10% fetal bovine serum (Gibco). PANC-1 cells were cultured in DMEM medium (Gibco) containing 10% FBS. HPDE cells were cultured in K-SFM medium (Gibco) containing 10% FBS and 1% epidermal growth factor. All cell lines were maintained at cell culture incubator with 37 °C and 5% CO2. The details of other cell lines are available in Additional file 1.

### Cell viability assay

Cell viability was measured with CCK-8 kit, followed the manufacturer. Briefly, cancer cells seeded in 96-well plates were either treated with Sophoridine at serial concentrations for 48 h, or were treated for various time points (0, 24, 48, or 72 h). After treatment for indicated time, CCK- 8 solution was added and incubated with cancer cells for 4 h. The percentages of cell survival was measured by SpectraMax190 microplate reader (Molecular Devices) based on the absorbance.

### Cell cycle and apoptosis analysis

Propidium iodide (PI) staining assay was used to analyze the cell cycle distribution. After exposed to different concentrations of Sophoridine for 48 h, cancer cells were harvested and fixed with 70% ethanol, followed by centrifugation (3000 rpm, 5 min), incubation with 100 mg/mL RNase in PBS for 30 min at 37 °C, and then staining with 50 mg/mL PI in PBS. The cell cycle distribution were analyzed by a Cell Lab Quanta SC flow cytometer (Beckman Coulter, USA). The Annexin V–FITC Apoptosis Detection Kit (BioVision) was used for the apoptosis analysis. Cells (5 × 10^5^) were exposed to different concentrations of Sophoridine for 48 h. After resuspended in 500 ml binding buffer, cells were incubated with Annexin V– fluorescein isothiocyanate (FITC; 5 ml) and PI (5 ml). After 30-min incubation, cells were analyzed by fluorescence-activated cell sorting (FACS) by flow cytometer (Becton Dickinson). Annexin V–FITC–stained only cells which indicated early apoptosis and cells with Annexin V–FITC- and PI double positive signals were combined for analysis.

### Colony formation assay

Five hundred cancer cells per well were seeded into 6-well plates, and then treated with Sophoridine at different concentrations for 48 h. After treatment, the cells were allowed to form cell colonies for another 7 days. Then cell colonies were fixed with 4% paraformaldehyde and stained with 0.1% crystal violet. After 3 times washing and air-dried, the stained colonies were counted and photographed under microscope (Leica, Germany).

### Hoechst staining

Hoechst 33,342 was used to identify the apoptotic cells based on the morphological changes in the nuclear assembly as previously described [16].

### Mitochondrial membrane potential (ΔΨm) assay

After 20 μM Sophoridine cells were stained with JC-1, which can indicates the change of mitochondrial membrane potential. The wavelengths of 490/540 nm was used. The mitochondrial membrane potential was indicated by the ratio between 590 nm and 540 nm (red signal and green signal, relatively).

### Western blot analysis

Miapaca-2 and PANC-1 cancer cells were treated with Sophoridine at different concentrations for 48 h, and then whole-cell lysates were prepared for western blot analysis as previously described [17].

### Detection of intracellular ROS

The peroxide-sensitive fluorescent probe DCFH-DA was used to measure the intracellular ROS levels. Briefly, cells were suspended in 1 mM DCFH-DA at 37 °C for 30 min. After incubation, cells were washed twice with PBS and re-suspended in PBS. ROS accumulation was detected with Calibur flow cytometry system at the wavelength 488/538 nm.

### Animal treatment with Sophoridine

BALB/c homozygous (nu/nu) nude mice (6 weeks, 18 g) were purchased from Shanghai SLAC Laboratory Animal (China). All animal experiments were followed the National Institutes of Health Guide for the Care and Use of Laboratory Animals, and the animal protocols were approved by the Institutional Animal Care and Use Committee of Shanghai University of Traditional Chinese Medicine. The mice were maintained in pathogen-free environment for one week after arrival. 2 × 10^6^ Miapca-2 cells in 100 μl PBS were inoculated in the right flank of nude mice. One week later, mice were randomly divided into 3 groups (7 mice/group). 3 groups received relatively an intraperitoneally (i.p.) injection of PBS as control, 20 mg/kg of Sophoridine, or 40 mg/kg of Sophoridine daily. After 3 weeks treatment, mice were sacrificed to weigh the tumors.

### Immunohistochemistry

Tumors were excised to 4-mm sections, fixed with formalin and embedded with paraffin. Slides were stained with antibodies against phospho-ERK, JNK, cleaved-caspase-3 and PCNA, then after washing stained with secondary antibody and visualized by the ChemMate EnVision Kit. The stained sections were analyzed under microscope at a magnification of × 400. Some sections were stained with H&E for the histological analysis.

### Statistical analysis

The data is presented as the mean ± S.D. Student’s t-test was used to determine the significance of the difference between two groups, and a *P* value <0.05 was considered to be statistically significant.

## Results

### Sophoridine shows an extensive tumor-killing effects and exhibits the most potent cytotoxicity to pancreatic cancer cells

**Fig. 1:**
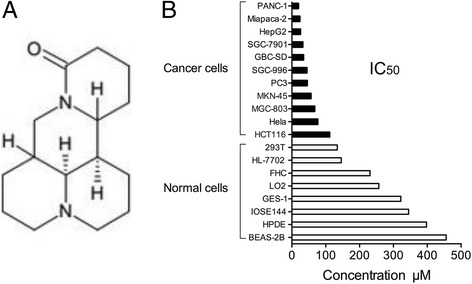
Selective killing effects of Sophoridine on cancer cells. **a** chemical structure of Sophoridine. **b** IC50 of Sophoridine for various cancer cells and normal cells. Normal cells, including human ovarian epithelial cells (IOSE144), human hepatic immortal cells (HL-7702 and LO2), immortalized human bronchial epithelial cells (BEAS-2B), human gastric epithelial cells (GES-1), human embryonic kidney (HEK) 293 T, human pancreatic ductal epithelial cell (HPDE), human colon epithelial cell (FHC), and a variety of human cancer cell lines, were treated with Sophoridine (0, 3.9, 7.8, 15.5, 31, 62.5, 125, 250, 500 μmol/L) for 48 h. Cytotoxicity was assessed with a CCK-8 assay, and IC50 values for Sophoridine on multiple cell lines were calculated by SPSS statistical software (SPSS Inc., Chicago, IL, USA). The results are representatives of at least 3 independent experiments

Sophoridine is a monomeric alkaloid extracted from sophora alopecuroides L (Fig. 1a). Modern pharmacologic evaluations have established that this herb medicine has potent cytotoxicity to cancer cells. We conducted a cell-based screening, examining the effects of Sophoridine on the cell viability of various tumor cells and normal cells (Fig. 1b, Additional file 2: Table S1). Eleven human cancer cell lines and 8 normal cell lines were exposed to various concentrations of Sophoridine (0–500 μmol/L) for 48 h. Cell viability was determined by the CCK-8 assay. Sophoridine exhibited remarkable inhibition effects to the growth of human pancreatic, gastric, liver, colon, gallbladder, and prostate carcinoma cells with IC50 values of about 20 μmol/L to 200 μmol/L. Among them, pancreatic cancer cells were the most sensitive cell lines to the cytotoxic effects of Sophoridine. Normal human pancreatic ductal epithelial cell (HPDE) and normal human bronchial epithelial cells (BEAS-2B) incubated with Sophoridine for 48 h, exhibited less sensitivity, indicating that Sophoridine selectively kills cancer cells.

### Sophoridine inhibits pancreatic cancer cell growth in both dose-dependent and time-dependent manners

**Fig. 2:**
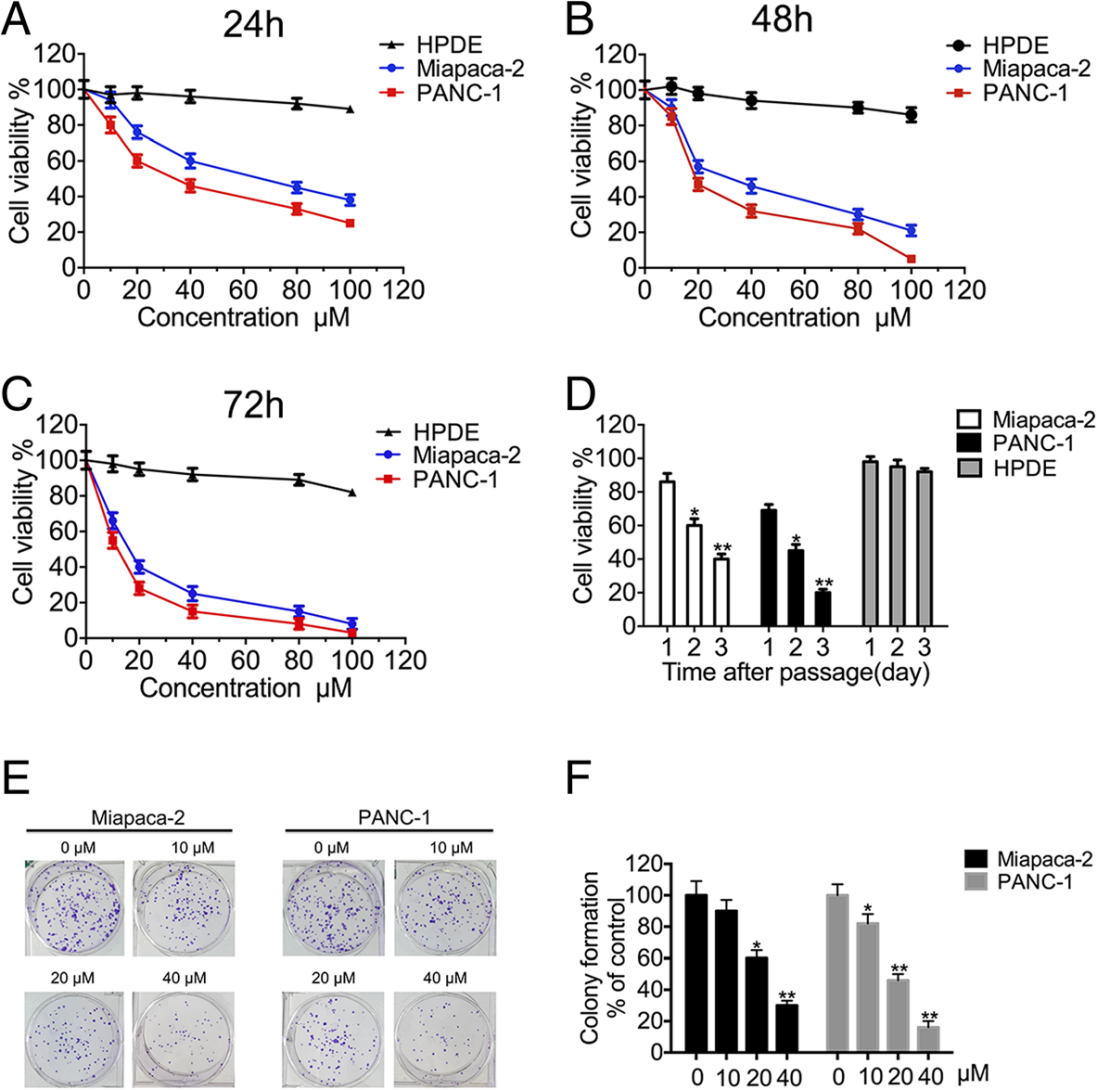
Sophoridine inhibits pancreatic cancer cell growth in both dose-dependent and time-dependent manners. **a**-**c** Two pancreatic cancer cells and one normal pancreatic ductal epithelial cell were assayed for cell viability after exposure to Sophoridine concentrations that ranged from 0 μM to 100 μM; cells treated with vehicle were set as controls. All the cells were cultured for 24 h, 48 h and 72 h. **d** At a fixed dose (20 μM), Sophoridine inhibited the viability of Pancreatic cancer cells and one normal pancreatic ductal epithelial cell in a time-dependent manner (24 h, 48 h, or 72 h). **e** Colony formation assays. Miapaca-2 and PANC-1 cells were treated with indicated doses of Sophoridine. The clones were visualized by crystal violet staining. **f** In the colony formation assay, the formed clones were counted manually for each group of cells. *, *P* < 0.05; **, *P* < 0.01

To evaluate the cytotoxic effects of Sophoridine on pancreatic cancer cells, two human pancreatic cancer cell lines (Miapaca-2 and PANC-1) and pancreatic ductal epithelial cell line (HPDE) were treated with Sophoridine at different concentrations (0, 10, 20, 40, 80, or 100 μM) for different lengths of time (24 h, 48 h, or 72 h). The CCK-8 assay showed that Sophoridine exhibited both concentration-dependent and time-dependent killing effects on multiple pancreatic cancer cell lines, but had no significant effect on HPDE cells (Fig. 2a-d), indicating that Sophoridine selectively kill cancer cells but not normal cells. Furthermore, we adopted the doses 0, 10, 20 and 40 μM of Sophoridine based on the initial cytotoxicity results and the concentrations used in the following colony formation assays (Fig. 2e). Quantitative analysis further revealed that colony numbers decreased with increased Sophoridine dosage (Fig. 2f). These data suggests that Sophoridine inhibits pancreatic cancer cell growth in both dose-dependent and time-dependent manners.

### Sophoridine induces S phase cell cycle arrest in pancreatic cancer cells

**Fig. 3:**
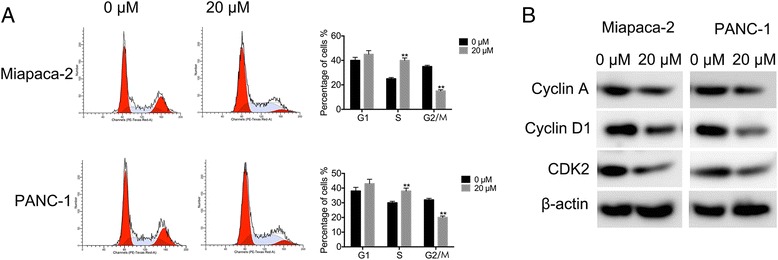
Sophoridine cause S phase cell cycle arrest in pancreatic cancer cells. **a** Pancreatic cancer cells were treated with or without 20 μM Sophoridine for the indicated time, and then flow cytometry was used to assess the cell cycle distribution. The percentage of each population was shown as mean ± S.D. Data is the representative of three independent experiments. *, *P* < 0.05; **, *P* < 0.01. **b** Western blotting analysis of G2/M transition-related proteins after Sophoridine treatment

In order to examine whether Sophoridine inhibits cell growth via cell cycle disturbance, cell cycle distribution and related cell cycle checkpoint factors were analyzed. We found that 20 μM Sophoridine treatment for 48 h to cancer cells leads to accumulated population in the S phase (Fig. 3a). Compared with the control, Sophoridine treatment increases S phase cell population from 26.23% (control) to 38.67% in Miapaca-2 cells and from 29.56% (control) to 39.16% in PANC-1 cells, about a 1.5-fold and a 1.3-fold increase, respectively. Additionally, Sophoridine treatment decreases the expression of Cyclin A, CDK2 and Cyclin D1 (Fig. 3b). In sum, S phase arrest contributes to the antiproliferative effects of Sophoridine in both cells and more significantly in Miapca-2 cells.

### Sophoridine induces mitochondrial-related apoptosis in pancreatic cancer cells

**Fig. 4:**
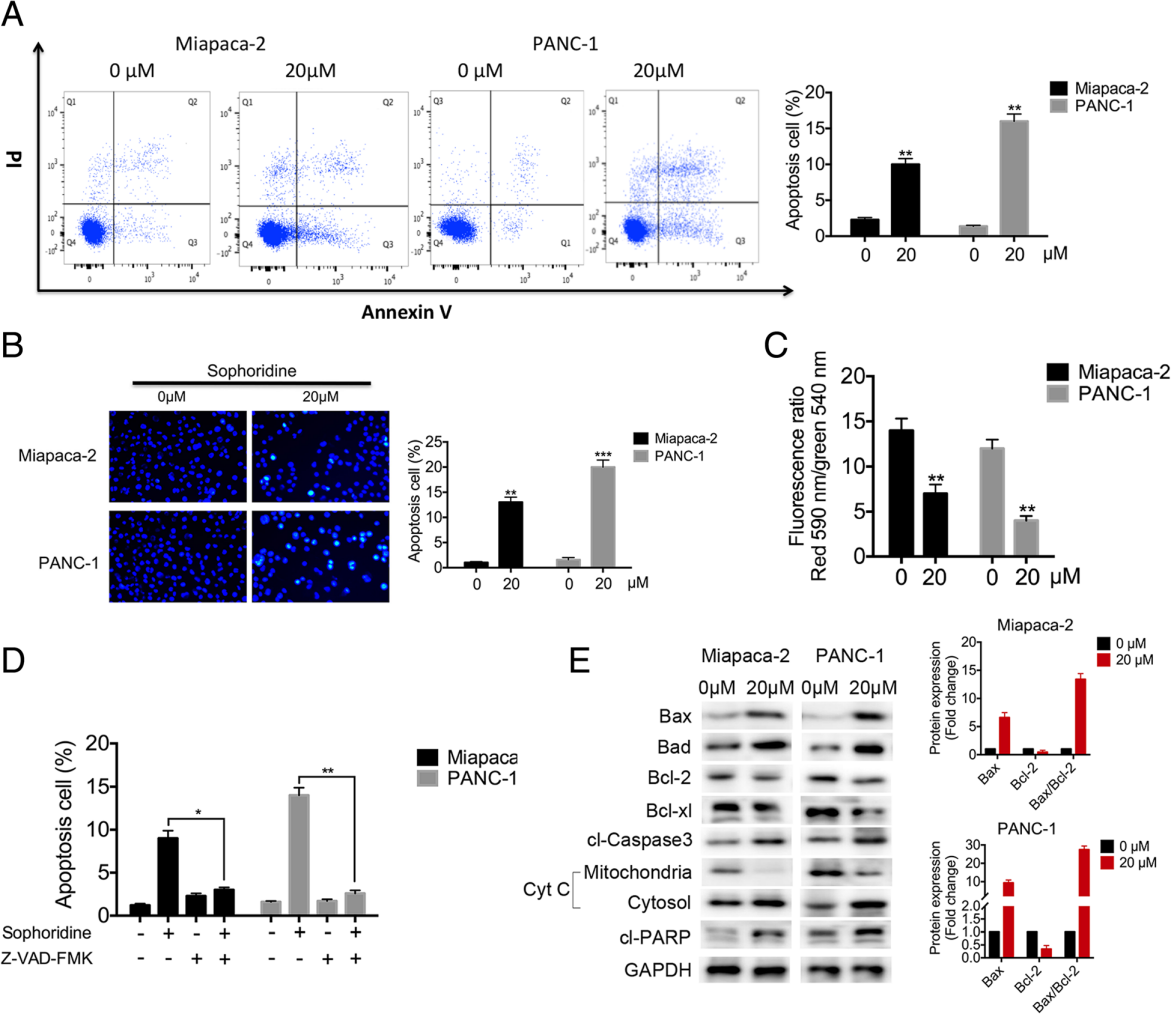
Sophoridine triggers mitochondrial-related apoptosis in pancreatic cancer cells. **a** Annexin V-FITC/PI assay and flow cytometry analysis were performed to assess apoptosis in Miapaca-2 and PANC-1 cells under 20 μM Sophoridine treatment for 48 h. Numbers indicate the percentage of cells in each quadrant. **b** DAPI staining was used to show the Sophoridine-induced morphological cellular changes. Representative photomicrographs of cells under control or 20 μM Sophoridine treatment are showed. **c** The mitochondrial membrane potential was detected by JC-1 staining and detected by flow cytometry. Decrease in the ratio of FITC signals to PE signals indicates the loss of ΔΨm. **d** After pretreated with 20 μM z-VAD-fmk, a pan caspase inhibitor, for 1 h, Miapaca-2 and PANC-1 cells were treated with 20 μM Sophoridine for 48 h. Apoptosis was evaluated by flow cytometry. **e** Western blotting analysis of bax, bad, bcl-2, bcl-xl, cleaved-caspase-3, cleaved-PARP and cytochrome c in cytosol and mitochondria after 20 μM Sophoridine treatment for the indicated time. Results are representatives of three separate experiments. GAPDH is set as control. *, *P* < 0.05; **, *P* < 0.01 versus the drug-untreated group

Next, the apoptotic effects of Sophoridine in pancreatic cancer cells were tested. Annexin V-FITC/PI double stainings were used to make a quantitative measurement of apoptosis. Twenty μM Sophoridine treatment for 48 h induces 10.65 ± 2.91% and 15.34 ± 2.36% apoptosis in Miapca-2 and PANC-1 cells, respectively (Fig. 4a). In addition, apoptotic cells indicated by nuclear condensation and fragmentation can be visualized by DAPI staining (Fig. 4b). We further tested the changes of the mitochondrial membrane potential (ΔΨm), bcl-2 family proteins, cytochrome c release, proteolytic cleavage of procaspase and the general caspase inhibitor (z-VAD-fmk) rescue assay. The loss of ΔΨm, measured by JC-1, can be measured after cells were exposed to 20 μM Sophoridine in both cell lines, especially in Miapaca-2 cells (Fig. 4c). To further test whether Sophoridine-induced cell apoptosis was caspase dependent, z-VAD-fmk, a pan caspase inhibitor, was used. As indicated in Fig. 4d and Additional file 3: Figure S1, the addition of z-VAD-fmk reduced the apoptotic population induced by Sophoridine from 10.89 to 3.73% in Miapaca-2 cells and from 14.79 to 2.32% in PANC-1 cells. The proto-oncoprotein Bcl-2 is a powerful antagonist of the mitochondrial pathway of apoptosis initiated by a variety of extra- and intracellular stresses. Therefore, we examined whether Sophoridine could alter the balance between proapoptotic Bax and antiapoptotic Bcl-2 proteins at the mitochondrial membrane. West blotting analysis showed that treatment of Miapca-2 and PANC-1 cells with 20 μM. Sophoridine significantly increased bad and bax levels, and decreased bcl-2 and bcl-xl levels in contrast, with a significant increase in Bax/Bcl-2 ratio. In consistent, cytochrome c was released, and cleavages of procaspase-3 and PARP were also observed (Fig. 4e). In addition, we found Sophoridine could upregulate ER stress mediators PERK and IRE but have no effect on ATF6 (Additional file 4: Figure S2). These results indicated that the activation of intrinsic apoptosis pathway was induced by Sophoridine.

### The activation of ERK and JNK mediates Sophoridine -induced S phase arrest and apoptosis

**Fig. 5:**
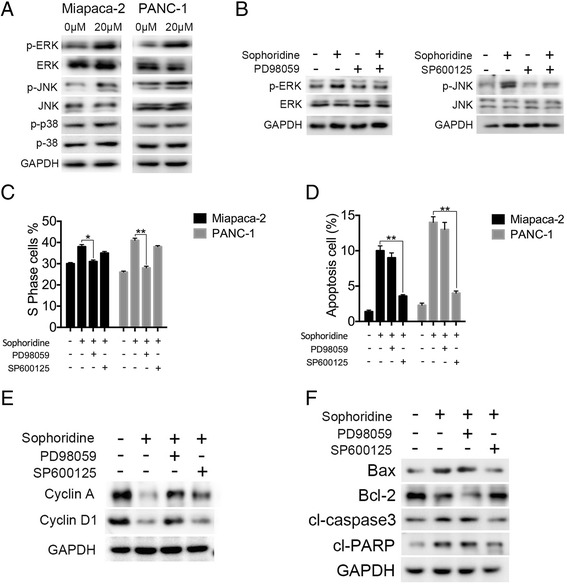
ERK and JNK activation mediated Sophoridine-induced S phase arrest and apoptosis, respectively. **a** After 20 μM Sophoridine treatment, the protein levels of total or phosphorylated MAPK members (JNK, p38, ERK) were detected by western blotting. **b** Cancer cells were pretreated with 20 mM MEK inhibitor (PD98059) and JNK inhibitor (SP600125) before the treatment with or without 20 μM Sophoridine for 48 h. The protein levels of total or phosphorylated ERK and JNK were detected by western blotting. **c**, **d** After pre-treated with the indicated inhibitor for 1 h, cancer cells were treated with 20 μM Sophoridine for 48 h. The cell cycle and cell apoptosis analysis was performed. **e**, **f** The indicated protein levels in treated cancer cells were assessed by western blotting

MAPK signaling pathway has been shown to play essential roles in the regulation of a wide variety of cellular processes, including cell growth, cell cycle regulation, migration, differentiation, development, and apoptosis [18]. To further study the underlying mechanisms of Sophoridine anti-tumor effects, the activations of ERK, JNK and p38 MAP kinases were evaluated in both cell lines. West blotting showed that the phosphorylation levels of ERK and JNK kinases were significantly increased after Sophoridine treatment, but the phosphorylation level of p38 was little affected (Fig. 5a). Pre-treatment with 20 μM PD98059 or SP600125 almost completely blocked the phosphorylation of ERK or JNK induced by Sophoridine (Fig. 5b). Interestingly, as shown in Fig. 5c and d and Additional file 5: Figure S3, only the ERK inhibitor PD98059 had a reversible effects on the S phase cell cycle arrest induced by Sophoridine and the JNK inhibitor SP600125 significantly decreased cell apoptosis in response to Sophoridine. Consistent with the results of flow cytometry, western blot revealed that PD98059 treatment had an apparently opposite effects compared with Sophoridine on the S transition-related protein levels, but it did not significantly affect the apoptosis-related proteins. In contrast, SP600125 decreased the Sophoridine-induced apoptosis proteins, but it did not affect S phase transition-related proteins (Fig. 5e and f). These data indicated that the effects of apoptosis and S phase arrest in Miapaca-2 cells induced by Sophoridine were mainly mediated by activation of the JNKand the ERK signaling pathway.

### ROS is required for Sophoridine-induced cell cycle arrest and apoptosis

**Fig. 6:**
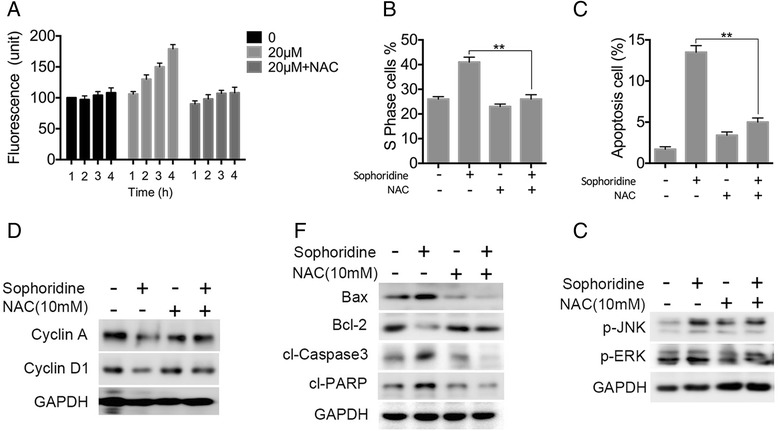
Sophoridine promotes the generation of ROS, which contributes to S phase cell cycle arrest and apoptosis in cancer cells. **a** Sophoridine in time-dependent manner upregulated ROS levels in Miapaca-2. After pretreated with 5 mM NAC for 2 h, cells were treated with or without 20 μM Sophoridine for indicated time, and then the intracellular levels of ROS were measured. **b**, **c** The treated cancer cells were sent to cell cycle and cell apoptosis analysis by FACS. **d**-**f** After pretreated with 5 mM NAC for 2 h, cells were treated with or without 20 μM Sophoridine for indicated time. The proteins of Cyclin A, Cyclin D1, Bcl-2, Bax, cleaved caspase-3, PARP, p-ERK and p-JNK in control or treated cancer cells were assessed by western blotting. **, *P* < 0.01 versus the NAC-untreated group

Since ROS generation is associated with the loss of mitochondrial membrane potential [19], we measured the levels of ROS in Miapaca-2 cells treated with Sophoridine. As shown in Fig. 6a, the levels of ROS in cells after Sophoridine treatment were increased in a time-dependent manner. Next, to test whether the increased ROS generation may have a role in Sophoridine-induced cell cycle arrest or apoptosis, we pretreated the cells with the antioxidant N-acetylcysteine (NAC) 1 h before 48-h Sophoridine treatment. The results showed that pretreatment with NAC leaded to a significant inhibition of the Sophoridine-induced cell cycle arrest and apoptosis (Fig. 6b and c and Additional file 6: Figure S4). In addition, pivotal proteins associated with the cell cycle transition and apoptosis were further test to validate the role of ROS in Sophoridine’s antineoplastic effects. Western blotting analysis revealed that NAC restored the expression of Cyclin A and Cyclin D1 (Fig. 6d). Similarly,NAC decreased the cleavage of caspase-3 and PARP, and the expression of bax, and increased the Bcl-2 expression (Fig. 6e). Moreover, the activation of ERK and JNK kinases in Miapaca-2 cells treated with Sophoridine can be attenuated by NAC (Fig. 6f). These data suggest that Sophoridine-induced ROS generation activates ERK and JNK kinases, which trigger cell cycle arrest and mitochondrial apoptotic pathways in pancreatic cancer cells.

### Sophoridine suppressed tumor growth in vivo

**Fig. 7:**
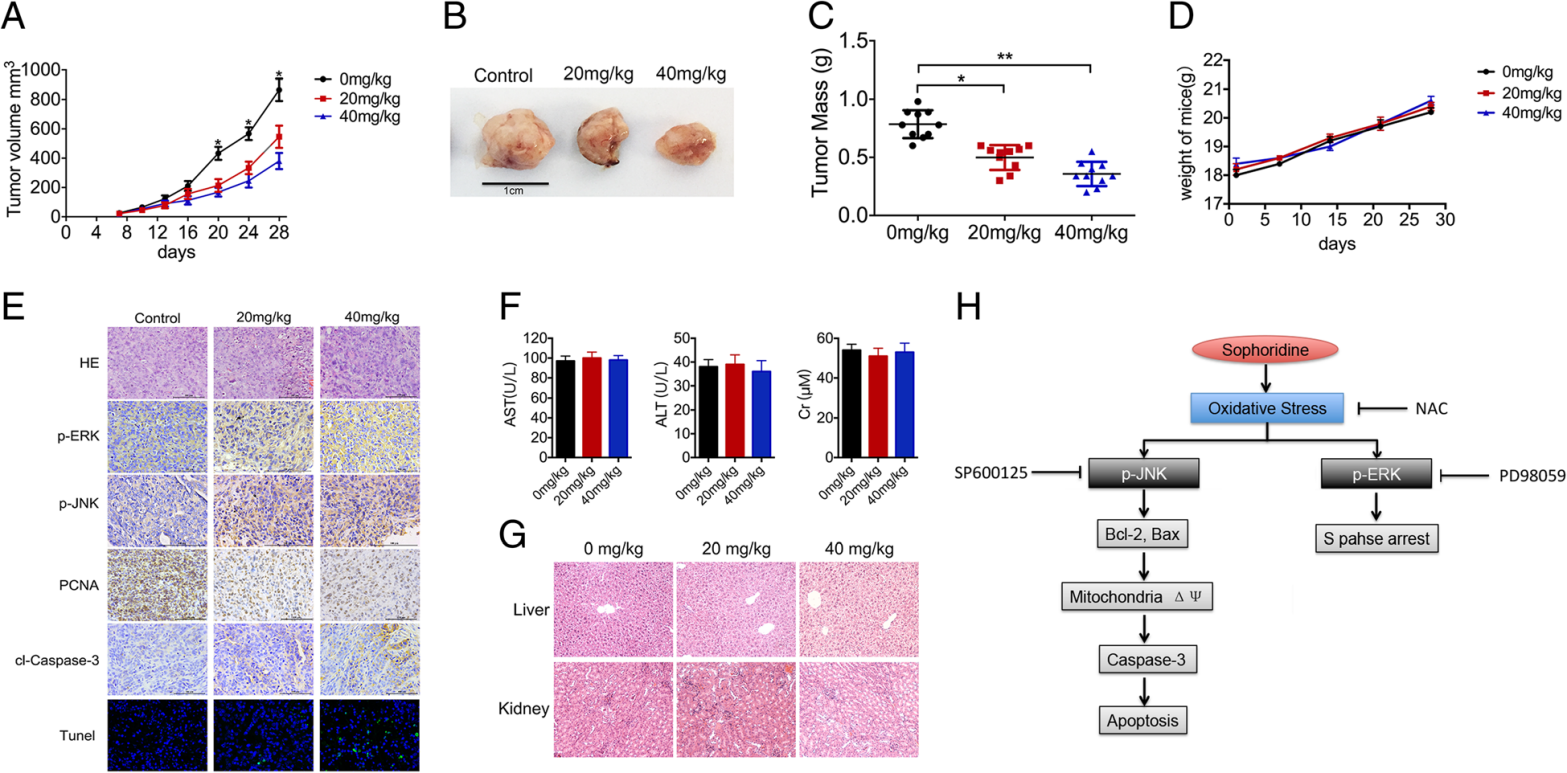
Sophoridine inhibits pancreatic cancer cell growth in vivo. **a** 2 × 10^6^ Miapaca-2 cells were inoculated into Balb/c nude mice. Mice were randomized into three groups (*n* = 7) and treated with PBS, or Sophoridine at the dose of 20 mg/kg or 40 mg/kg daily for 3 weeks. Tumor volumes were measured by digital caliper every 3 days. **b** The tumors were excised from mice after last treatment. **c** Tumor masses were weighed and compared, and each histogram represented the mean ± S.D. **d** The body weight was measured every week. **e** The phosphorylation signals of p-ERK, p-JNK and PCNA in xenograft tumor tissues were detected by immunohistochemistry. Apoptosis cells in mice tumor were detected by Tunel staining (**f**) The liver and kidney biochemical functions were evaluated. The AST, ALT and Cr levels of mouse blood were detected by Elisa assay. **g** The liver and kidney from control and Sophoridine group were stained with H&E to evaluate the toxicity after treatment. The histological structure of liver and kidney were observed and compared microscopically. **h** Overview of signaling pathways for Sophoridine-induced cell cycle arrest and apoptosis in human pancreatic tumors

To further validate Sophoridine can inhibit tumor growth in vivo, 2 × 10^6^ Miapaca-2 cells were subcutaneously inoculated in Balb/c nude mice. Sophoridine treatment was administrated from the 7th day at 20 or 40 mg/kg intraperitoneally for 21 days. We found that Sophoridine showed significant inhibitory effects on tumor volume (Fig. 7a and b). The mass of tumors under 20 or 40 mg/kg Sophoridine treatment was significantly less than that of the control group (Fig. 7c). However, there was no significant changes on body weight under Sophoridine treatment (Fig. 7d). Furthermore, the activation of ERK and JNK in xenograft tumor tissues was tested by immunohistochemistry and immunoblotting. It showed that both ERK and JNK were activated in Sophoridine-treated xenograft tumor tissues (Fig. 7e and Additional file 7: Figure S5). In addition, IHC analysis of proliferating cell nuclear antigen (PCNA), cleaved caspase-3 and tunel staining showed significantly fewer proliferative cells, and more apoptotic cells in Sophoridine-treated tumors (Fig. 7e). The liver and kidney biochemical functions were monitored, and there were no obvious difference between control and Sophoridine treatment group (Fig. 7f). The liver and kidney from control and Sophoridine group were stained with H&E to further evaluate the toxicity after treatment. The histological structure of liver and kidney were observed and compared microscopically, and there were almost no histological changes after Sophoridine treatment (Fig. 7g). These results suggest that Sophoridine was an effective agent that can inhibit the growth of xenograft pancreatic tumors in vivo with well-tolerated toxicity.

## Discussion

Conventional chemotherapy remains the mainstay in human cancer treatment, however the response rates to most chemotherapeutic agents are still low, and the clinical benefits are marginal. In addition, its severe toxicities and drug resistance reduce patients’ life quality and limit the effective application of these agents. Because of their rich structural diversity and promising therapeutic applications, natural products and their derivatives have recently caught the attention of pharmacologists and chemists [4, 5]. Our study focus on developing novel, effective, and safe drugs from natural products for cancer therapy [16, 17]. In the present study, we showed that Sophoridine can selectively kill pancreatic cancer cell without harming the normal cells. Furthermore, we found that Sophoridine significantly suppressed tumor growth in vitro and in vivo. The compound triggered S phase cell cycle arrest and induced cell apoptosis mainly via MAPK signaling pathways.

MAPKs, including ERK, JNK, and p38 are main mediators of cellular responses to extracellular signals. MAPK signaling plays a critical role in chemical-triggered cell cycle arrest and apoptotic processes. ERK is generally involved in proliferation and growth regulation. The activation of JNK and p38 MAPKs are generally induced by stress and closely associated with cell death [20]. To demonstrate the detailed mechanisms underlying the Sophoridine-induced cell cycle arrest and cell death, we tested the effects of Sophoridine on MAPK signaling activation. We found that Sophoridine treatment substantially activated ERK and JNK in pancreatic cancer cells, and the phosphorylation levels were dependent on ROS levels, supporting by the data that the activation was abrogated by addition of NAC. Besides, Sophoridine induced Bcl-2/Bax modulation, caspase-3 activation, PARP1 cleavage and consequent apoptosis mainly via JNK activation since JNK inhibitor SP600125 treatment can inhibit the apoptotic events. In addition, ERK activation was responsible for Sophoridine-induced cell cycle arrest, which was reversed by ERK inhibitor PD98059 treatment. These results suggest that ERK and JNK activation mediated Sophoridine-induced cell cycle arrest and cell apoptosis respectively.

ROS exists in all aerobic cells in balance with antioxidants. When excess ROS production and/or antioxidant depletion occur, the balance would be disrupted and oxidative stress would be generated [21]. Accumulating evidence indicates that many chemotherapeutic agents are selectively toxic to tumor cells because they increase the oxidant stress to the extent that is beyond cancer cell’s limit [22–24]. The intrinsic apoptosis triggered cytotoxic ROS through activation of MAPK pathways. Previous studies also showed that ROS is involved in the diallyl tri-sulfide induced cell cycle arrest in human prostate cancer cells [25]. Here we found that accumulation of ROS is critical to Sophoridine-induced cell cycle arrest and cell apoptosis, supported by the evidence that pretreatment with the inhibitor NAC partially prevented Sophoridine-induced cell cycle arrest and cell apoptosis (Fig. 7h).

## Conclusion

The present study demonstrated the molecular mechanisms of the antitumor effects of Sophoridine in human pancreatic tumors. Sophoridine can significantly induce mitochondrial-related apoptosis via the JNK signaling and induce S phase cell cycle arrest through the ERK signaling. Moreover, ROS is required for Sophoridine-induced cell cycle arrest and apoptosis. Additionally, Sophoridine can significantly inhibit the growth of pancreatic cancer in vitro *and* in vivo. Thus, Sophoridine as a potential anti-cancer agent, is promising to be a new, effective therapy for pancreatic cancer.

## Additional files

Additional file 1: Supplementary Material and Method. (DOCX 46 kb)

Additional file 2: Table S1. IC50 values of Sophoridine for various cancer cells and normal cells. (DOCX 63 kb)

Additional file 3: Figure S1. After pretreated with 20 μM z-VAD-fmk, a pan caspase inhibitor, for 1 h, Miapaca-S2 and PANC-1 cells were treated with 20 μM Sophoridine for 48 h. Apoptosis was evaluated by flow cytometry. (JPEG 537 kb)

Additional file 4: Figure S2. After 20 μM Sophoridine treatment, the protein levels of total or phosphorylated IRE, PERK and ATF6 were detected by western blotting. (JPEG 90 kb)

Additional file 5: Figure S3. After pre-treated with the indicated inhibitor for 1 h, cancer cells were treated with 20 μM Sophoridine for 48 h. The cell cycle and cell apoptosis analysis was performed. (JPEG 1426 kb)

Additional file 6: Figure S4. After pretreated with 5 mM NAC for 2 h, cells were treated with or without 20 μM Sophoridine for indicated time, and then the treated cancer cells were sent to cell cycle and cell apoptosis analysis by FACS. (JPEG 571 kb)

Additional file 7: Figure S5. The p-ERK and p-JNK expression were detected by western blot in mice tumor treated with Sophoridine. (JPEG 45 kb)

## Abbreviations

CCK-8: Cell Counting Kit-8
HPDE: human pancreatic ductal epithelial cell
NAC: N-acetylcysteine
PCNA: Proliferating cell nuclear antigen
PI: Propidium iodide
ΔΨm: Mitochondrial membrane potential
