# Correction to: Sophoridine induces apoptosis and S phase arrest via ROS-dependent JNK and ERK activation in human pancreatic cancer cells

**DOI:** 10.1186/s13046-020-01764-y

**Published:** 2020-11-26

**Authors:** Zihang Xu, Fei Zhang, Chao Bai, Chao Yao, Hairong Zhong, Chunpu Zou, Xiao Chen

**Affiliations:** 1 School of Basic Medical Science, Shanghai University of Traditional Chinese Medicine, Shanghai, 201203 China; 2 Department of General Surgery, Xinhua Hospital affiliated to Shanghai Jiao Tong University School of Medicine, Shanghai, 200092 China; 3 Department of general surgery, First Hospital of Shanxi Medical University, No. 85 South of Jiefang road, Taiyuan, 030001 China

**Correction to: J Exp Clin Cancer Res 36, 124 (2017)**

**https://doi.org/10.1186/s13046-017-0590-5**

Following publication of the article [1], the authors identified errors in Fig. 7; specifically panels in Fig. 7g (H&E staining of liver and kidney from the control and Sophoridine group). The corrected figure can be found below. The corrections do not change the results or the conclusions of this paper.

**Fig. 7:**
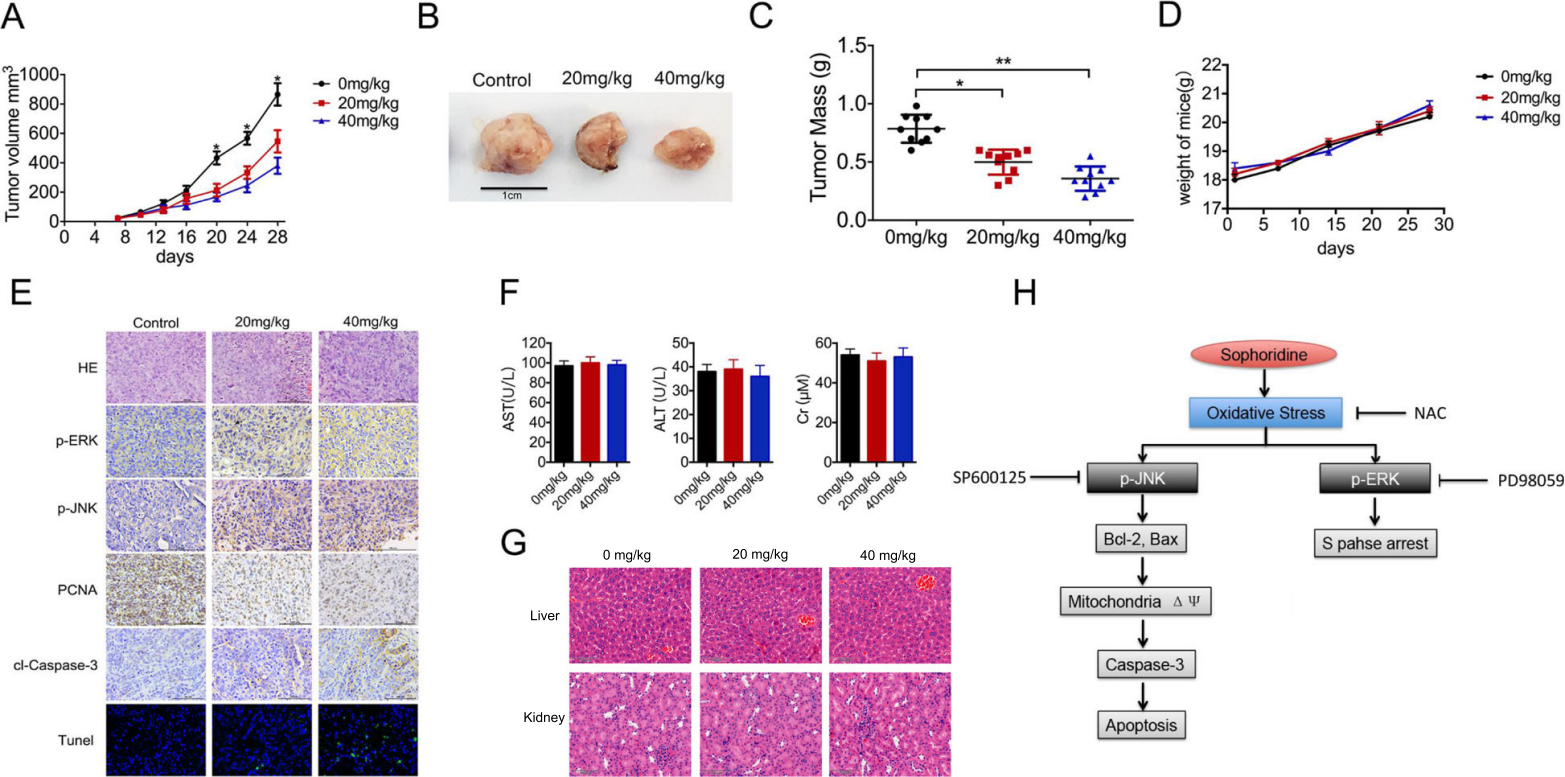
Sophoridine inhibits pancreatic cancer cell growth in vivo. **a** 2 × 10^6^ Miapaca-2 cells were inoculated into Balb/c nude mice. Mice were randomized into three groups (*n* = 7) and treated with PBS, or Sophoridine at the dose of 20 mg/kg or 40 mg/kg daily for 3 weeks. Tumor volumes were measured by digital caliper every 3 days. **b** The tumors were excised from mice after last treatment. **c** Tumor masses were weighed and compared, and each histogram represented the mean ± S.D. **d** The body weight was measured every week. **e** The phosphorylation signals of p-ERK, p-JNK and PCNA in xenograft tumor tissues were detected by immunohistochemistry. Apoptosis cells in mice tumor were detected by Tunel staining (**f**) The liver and kidney biochemical functions were evaluated. The AST, ALT and Cr levels of mouse blood were detected by Elisa assay. **g** The liver and kidney from control and Sophoridine group were stained with H&E to evaluate the toxicity after treatment. The histological structure of liver and kidney were observed and compared microscopically. **h** Overview of signaling pathways for Sophoridine-induced cell cycle arrest and apoptosis in human pancreatic tumors

The original article has been updated.

## References

[1] Xu Z (2017). Sophoridine induces apoptosis and S phase arrest via ROS-dependent JNK and ERK activation in human pancreatic cancer cells. J Exp Clin Cancer Res.

