# Supplementary material for: Sophoridine induces apoptosis and S phase arrest via ROS-dependent JNK and ERK activation in human pancreatic cancer cells
Source: J Exp Clin Cancer Res. 2017 Sep 11;36:124. doi: 10.1186/s13046-017-0590-5 (PMC5594456; doi:10.1186/s13046-017-0590-5)
Supplement: Supplementary file 1 — Supplementary Material and Method. (DOCX 46 kb) [file 13046_2017_590_MOESM1_ESM.docx]

HepG2, SGC-7901, SGC-996, PC3, cells were maintained in RPMI-1640 (Gibco, NY, USA) containing with 100 U/mL penicillin-streptomycin (Hyclone, UT, USA) and 10% fetal bovine serum (Gibco). GBC-SD, MKN45, MGC-803, Hela, HCT116, 293T, LO2, HL-7702, cells were cultured in DMEM medium (Gibco) containing 10% FBS. FHC, GES-1, IOSE144, BEAS-2B cells were cultured in DMEM medium (Gibco) containing 10% FBS and 1% epidermal growth factor. All cell lines were maintained at cell culture incubator with 37°C and 5% CO_2_.
