# Supplementary material for: Sophoridine induces apoptosis and S phase arrest via ROS-dependent JNK and ERK activation in human pancreatic cancer cells
Source: J Exp Clin Cancer Res. 2017 Sep 11;36:124. doi: 10.1186/s13046-017-0590-5 (PMC5594456; doi:10.1186/s13046-017-0590-5)
Supplement: Supplementary file 2 — IC50 values of Sophoridine for various cancer cells and normal cells. (DOCX 63 kb) [file 13046_2017_590_MOESM2_ESM.docx]

| **Cell lines** | **Sophoridine μM（IC50）** |
| --- | --- |
| PANC-1 | 19.23 |
| Miapaca-2 | 22.65 |
| HepG2 | 26.78 |
| SGC-7901 | 32.56 |
| GBC-SD | 34.67 |
| SGC-996 | 45.70 |
| PC-3 | 46.37 |
| MKN-45 | 65.38 |
| MGC-803 | 74.68 |
| Hela | 85.16 |
| HCT116 | 105.67 |
| 293T | 137.64 |
| HL-7702 | 146.84 |
| FHC | 229.46 |
| LO2 | 261.35 |
| GES-1 | 325.46 |
| IOSE114 | 342.57 |
| HPDE | 402.34 |
| BEAS-2B | 458.73 |

Table1 IC50 of Sophoridine for various cancer cells and normal cells.
