# Supplementary figures and images for: Sophoridine induces apoptosis and S phase arrest via ROS-dependent JNK and ERK activation in human pancreatic cancer cells
Source: J Exp Clin Cancer Res. 2017 Sep 11;36:124. doi: 10.1186/s13046-017-0590-5 (PMC5594456; doi:10.1186/s13046-017-0590-5)

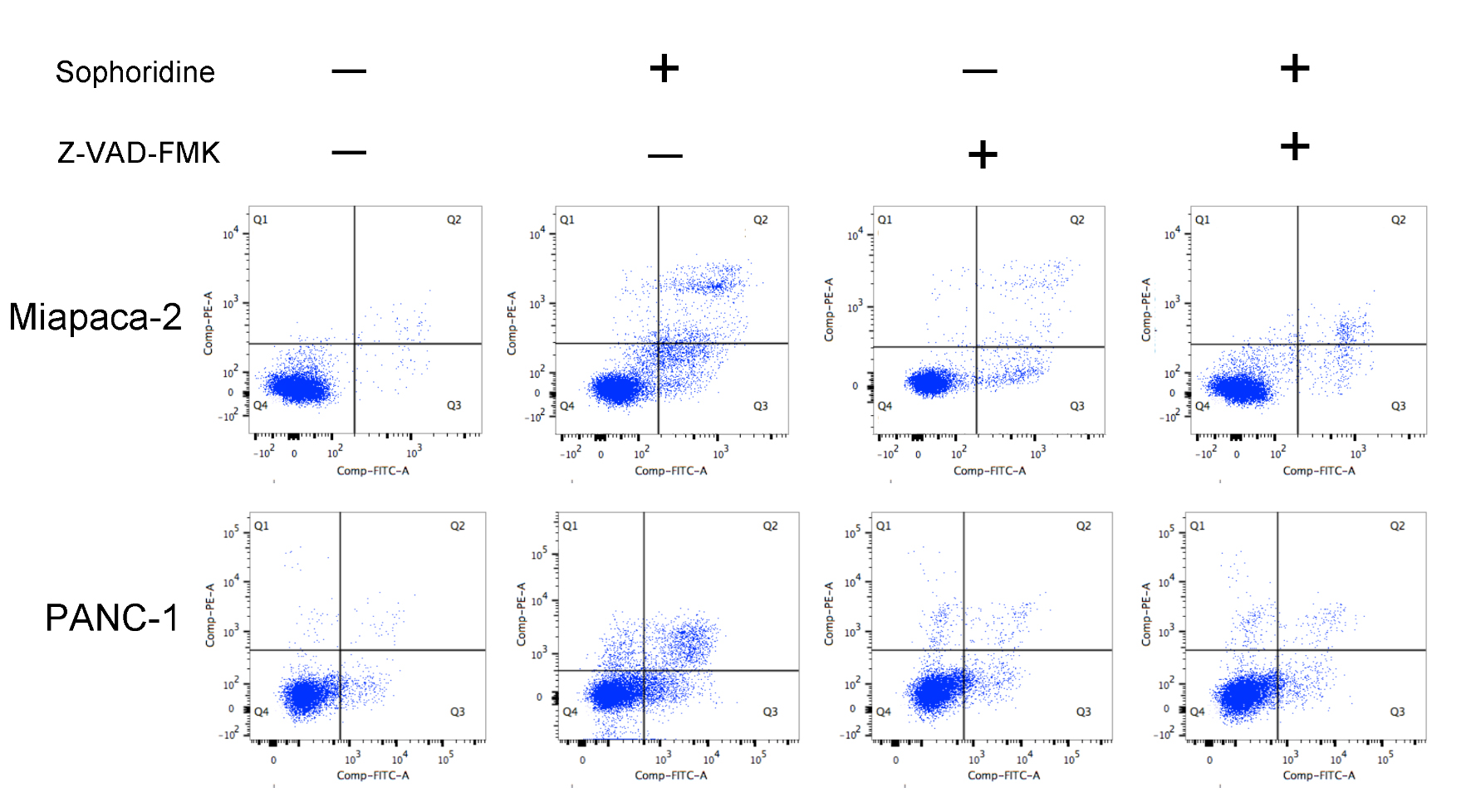

Supplement: Supplementary file 3 — After pretreated with 20 μM z-VAD-fmk, a pan caspase inhibitor, for 1 h, Miapaca-S2 and PANC-1 cells were treated with 20 μM Sophoridine for 48 h. Apoptosis was evaluated by flow cytometry. (JPEG 537 kb) [file 13046_2017_590_MOESM3_ESM.jpg]

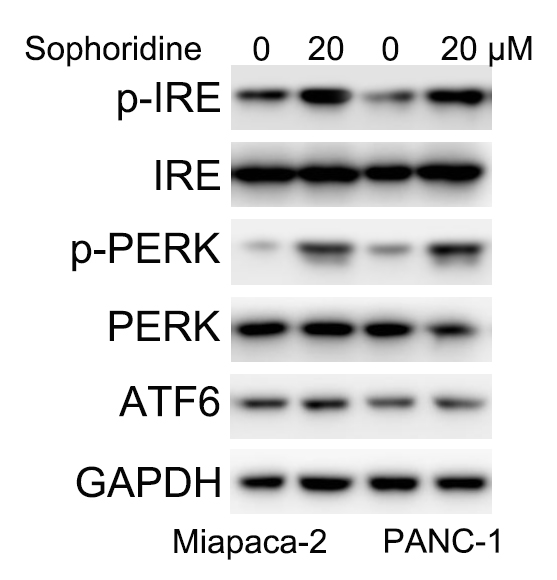

Supplement: Supplementary file 4 — After 20 μM Sophoridine treatment, the protein levels of total or phosphorylated IRE, PERK and ATF6 were detected by western blotting. (JPEG 90 kb) [file 13046_2017_590_MOESM4_ESM.jpg]

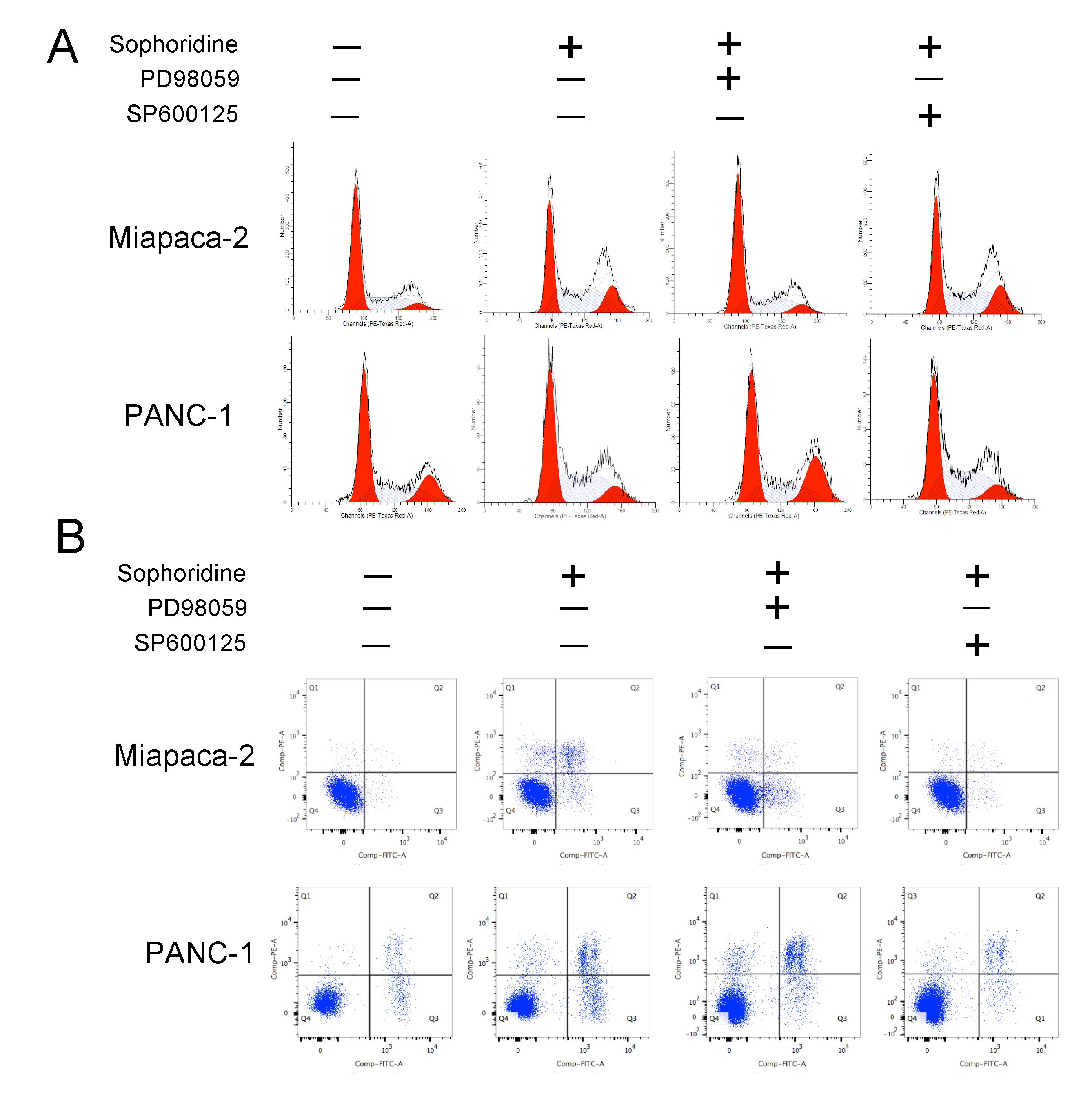

Supplement: Supplementary file 5 — After pre-treated with the indicated inhibitor for 1 h, cancer cells were treated with 20 μM Sophoridine for 48 h. The cell cycle and cell apoptosis analysis was performed. (JPEG 1426 kb) [file 13046_2017_590_MOESM5_ESM.jpg]

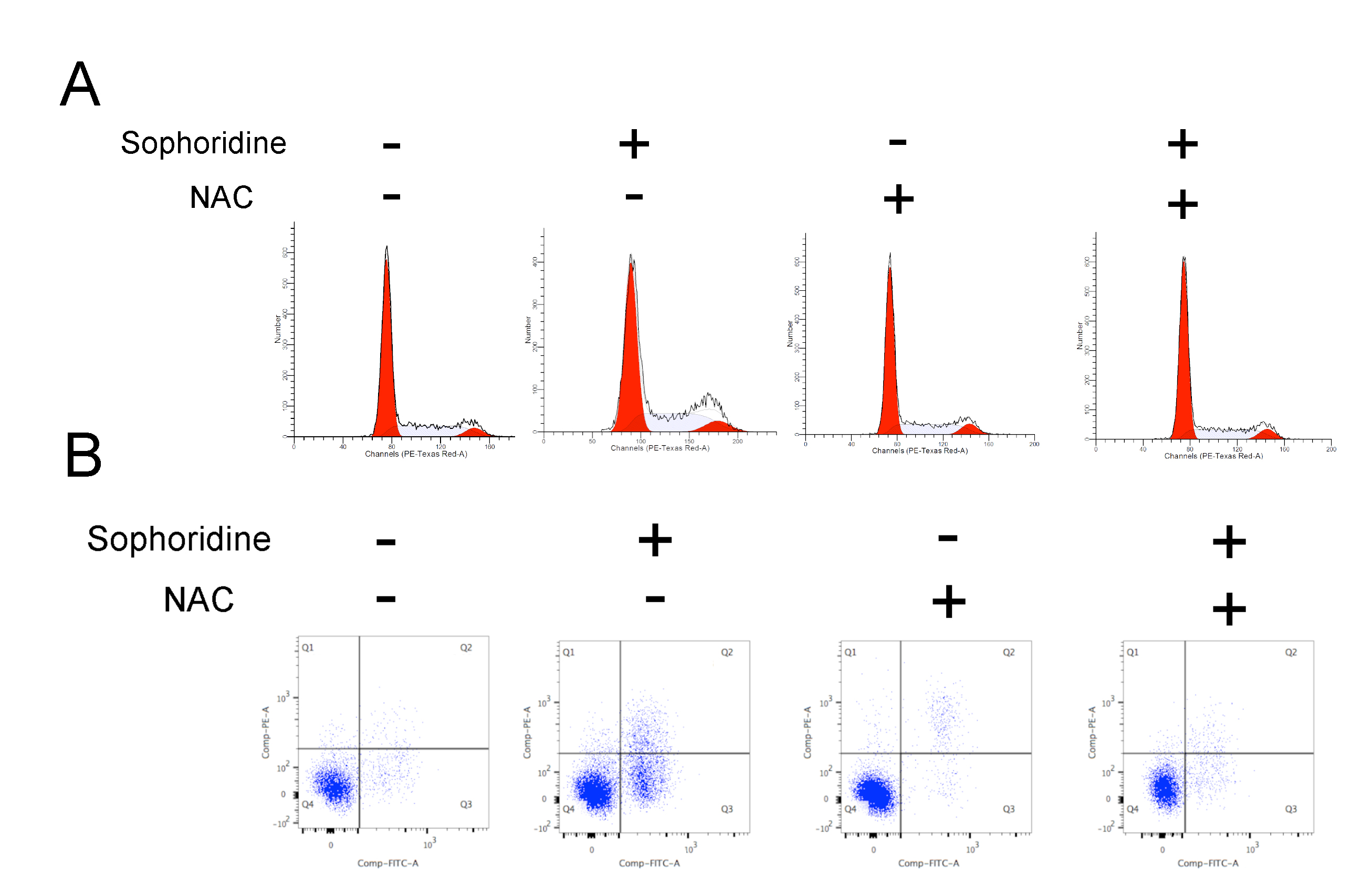

Supplement: Supplementary file 6 — After pretreated with 5 mM NAC for 2 h, cells were treated with or without 20 μM Sophoridine for indicated time, and then the treated cancer cells were sent to cell cycle and cell apoptosis analysis by FACS. (JPEG 571 kb) [file 13046_2017_590_MOESM6_ESM.jpg]

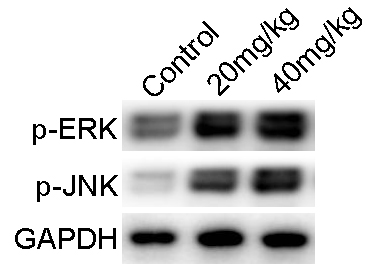

Supplement: Supplementary file 7 — The p-ERK and p-JNK expression were detected by western blot in mice tumor treated with Sophoridine. (JPEG 45 kb) [file 13046_2017_590_MOESM7_ESM.jpg]
